# Mutational analysis of *TSC1* and *TSC2* in Danish patients with tuberous sclerosis complex

**DOI:** 10.1038/s41598-020-66588-4

**Published:** 2020-06-18

**Authors:** Thomas Rosengren, Santoesha Nanhoe, Luis Gustavo Dufner de Almeida, Bitten Schönewolf-Greulich, Lasse Jonsgaard Larsen, Caroline Amalie Brunbjerg Hey, Morten Dunø, Jakob Ek, Lotte Risom, Mark Nellist, Lisbeth Birk Møller

**Affiliations:** 1 Clinical Genetics Clinic, Copenhagen University Hospital, Rigshospitalet. Address 1: Kennedy Center, Gl landevej 7, DK-2600, Glostrup, Denmark. Address 2: 4062, Blegdamsvej 9, DK-2100 Østerbro, Denmark; 2 Department of Clinical Genetics, Erasmus Medical Center, Rotterdam, The Netherlands; 3 Department of Genetics and Evolutionary Biology, Institute of Biosciences, University of Sao Paulo, Sao Paulo, Brazil

**Keywords:** Diseases, Cancer

## Abstract

Tuberous sclerosis complex (TSC) is an autosomal dominant disorder characterized by hamartomas in the skin and other organs, including brain, heart, lung, kidney and bones. TSC is caused by mutations in *TSC1* and *TSC2*. Here, we present the *TSC1* and *TSC2* variants identified in 168 Danish individuals out of a cohort of 327 individuals suspected of TSC. A total of 137 predicted pathogenic or likely pathogenic variants were identified: 33 different *TSC1* variants in 42 patients, and 104 different *TSC2* variants in 126 patients. In 40 cases (24%), the identified predicted pathogenic variant had not been described previously. In total, 33 novel variants in *TSC2* and 7 novel variants in *TSC1* were identified. To assist in the classification of 11 *TSC2* variants, we investigated the effects of these variants in an *in vitro* functional assay. Based on the functional results, as well as population and genetic data, we classified 8 variants as likely to be pathogenic and 3 as likely to be benign.

## Introduction

Tuberous sclerosis complex (TSC) is an autosomal dominant disorder of high penetrance with an incidence of 1:6,000–1:10,000 and an estimated prevalence of 1:14,000–1:25,000^1,2^. TSC is characterized by the presence of mainly benign tumors that can affect multiple organ systems e.g. the central nervous system, heart, kidney, lung, bone and skin. TSC patients are phenotypically and genetically heterogeneous and there is considerable variation in the number, location and size of the different TSC-associated lesions. Mutations in one of two genes, *TSC1* (OMIM#191100) and *TSC2* (OMIM#191092), cause TSC^3,4^.

**Figure 1:**
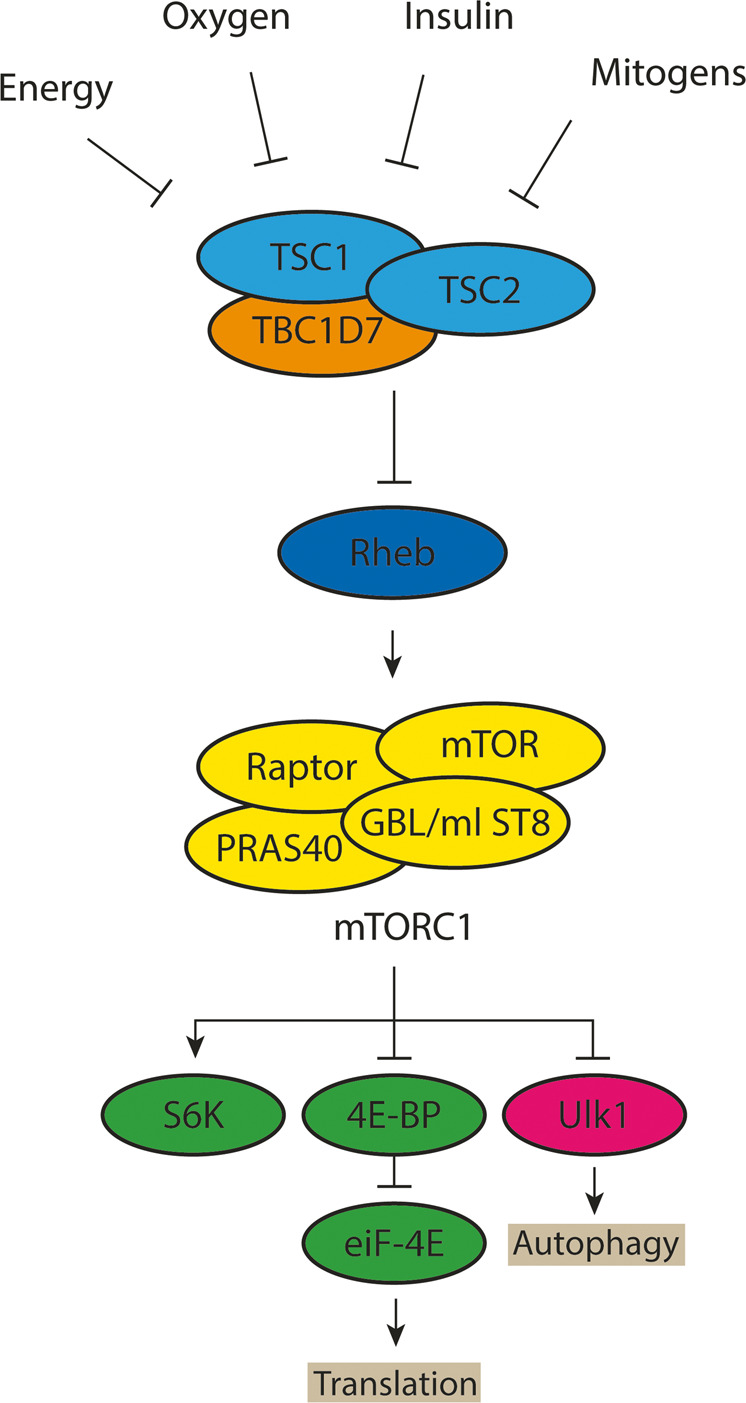
Tuberous Sclerosis Complex signaling. The TSC complex is a central node in mTORC1 signaling and receives inputs from multiple cellular pathways that influencing TSC complex activity. mTORC1 also responds to amino acids through the RAG GTPases (not shown). However, the amino acid dependent regulation of mTORC1 is independent of the TSC complex. Inhibitory and activating phosphorylation events are indicated.

*TSC1* is located on chromosome 9q34 and consists of 23 exons, which encode the 130 kDa TSC1 protein, hamartin. *TSC2* is located on chromosome 16p13.3 and consists of 42 exons which encode the 200 kDa TSC2 protein, tuberin. TSC1 and TSC2, together with a third subunit, TBC1D7^5^, form a stable protein complex, the TSC complex. The TSC complex is a GTPase-activating protein (GAP) specific for the small GTPase, Ras homologue enriched in brain (RHEB)^6^. Active RHEB is involved in the activation of the mechanistic target of rapamycin (mTOR) complex 1 (mTORC1), a critical regulator of anabolic processes such as protein and lipid synthesis^7^. The TSC complex inactivates RHEB to down-regulate mTORC1 signaling and inhibit cell growth. TSC-associated tumors are characterized by increased phosphorylation of S6, elongation factor 4E binding protein 1 (4E-BP1), p70 S6 kinase (S6K) and other downstream targets of mTORC1 (Fig. 1).

Approximately 2/3 of TSC cases are due to sporadic *de novo* germline mutations^2^. *TSC2* mutations are identified in the majority of TSC patients and, in general, cause a more severe phenotype than *TSC1* mutations^8,9^. Exceptions to this rule are however observed^10,11^.

Large genomic deletions that affect both *TSC2* and the adjacent *PKD1* (OMIM# 601313) locus are associated with a subset of patients with TSC and severe, early-onset autosomal dominant polycystic kidney disease.

While a pathogenic *TSC1* or *TSC2* variant can be identified in most TSC patients, in 10–15% of affected individuals conventional molecular testing fails to identify the causative mutation. Recent studies indicate that this is most likely because these individuals are either mosaic for a pathogenic *TSC1* or *TSC2* variant, or have a pathogenic variant in a region of *TSC1* or *TSC2* that is not routinely screened^12–14^. In addition, it is not always clear whether an identified *TSC1* or *TSC2* variant is disease-causing. In such cases, functional assessment can help establish pathogenicity^15^.

In this report, we present the molecular test results of a cohort of 327 Danish patients suspected of TSC. Furthermore, the effects of eleven variants on the ability of the TSC complex to inhibit mTORC1 activity, were investigated using an *in vitro* functional assay.

## Material and methods

### Subjects

The project was performed according to the Declaration of Helsinki. Agreement was obtained from all the participants or, if under 18, from a parent, prior to molecular genetic testing. Between 2003 and 2018, 327 individuals suspected of TSC were identified in pediatric and clinical genetic departments in Denmark and referred to Copenhagen University Hospital for molecular diagnosis. Some patients fulfilled the clinical criteria for definite TSC^16^, whereas others only had one of the major features of TSC. In a large number of patients (approximately 80%) only very limited clinical information was provided. A total of 6 prenatal cases in which rhabdomyomas were revealed by ultrasound scanning were also included. Genomic DNA was prepared by standard methods from peripheral blood, or tissue, as described previously^17^.

### Screening for pathogenic variants

Screening for mutations in *TSC1* and *TSC2* was performed either by denaturing gradient gel electrophoresis (DGGE) (before 2006) as described previously^17^, by direct Sanger sequencing of PCR products of all coding exons plus 20 bp of flanking intronic sequences (in the period 2006–2017), or since 2017, by Next Generation Sequencing (NGS) on a MiSeq Benchtop Sequencer (Illumina) following HaloPlex Custom Region Enrichment (Agilent). NGS data was analyzed in SureCall software (Agilent) using a BWA MEM aligner and SNPPET SNP caller. At least 99% of the target region (exon sequences as well as 20 base pairs of flanking intron sequences) had a read depth >20. Variants identified by DGGE or NGS and selected for reporting were verified by Sanger sequencing. The primers used for PCR amplification of the individual exons are listed in Supplementary Tables 1 and 2. Single and multiple exon deletions and duplications were detected by multiplex ligation probe amplification (MLPA) using the SALSA MLPA P124-TSC1 and P046-TSC2 probe-mixes (MRC Holland).

### Nomenclature

The nomenclature of the identified mutations is given according to HGVS (www.hgvs.org) guidelines. Nucleotide numbering for *TSC1* is according to reference transcript NM_000368.4, and for *TSC2* according to NM_000548.3. In both cases, c.1 is the A of the ATG translation initiation codon, and p.M1 is the initiation codon. Genomic reference sequences were NG_012386.1 for *TSC1* and NG_005895.1 for *TSC2*. *TSC1* contains two non-coding exons (exon 1 and exon 2). *TSC2* contains one non-coding exon (exon 1).

### Functional investigation

We derived expression constructs for *TSC2* variants by site-directed mutagenesis (SDM) of a wild-type *TSC2* expression construct^18^. All constructs were verified by sequencing of the complete *TSC2* open reading frame. Each variant was tested in at least 3 separate transfection experiments in 3H9-1B1 (*TSC2*:/*TSC1* double knockout HEK 293 T) cells^19^. Cells expressing the variants were compared to cells expressing wild-type *TSC2*, the pathogenic *TSC2* p.Arg611Gln variant, and cells not expressing *TSC2* (*TSC1/S6K* only). A *S6K* reporter and *TSC1* expression constructs (both encoding a myc epitope tag) were included in each transfection mixture. Transfections were performed in 24-well dishes (1 × 10^5^–2 × 10^5^ cells per well). Cells were lysed 18 hours after transfection in 50 mM Tris-HCl (pH 7.6), 100 mM NaCl, 50 mM NaF, 1% Triton-X-100, 1 mM EDTA and Complete protease inhibitors (Roche, Basel, Switzerland). After centrifugation (10 000 x g for 10 minutes at 4 °C), the cleared cell lysates were separated by SDS-PAGE and transferred to nitrocellulose filters. The levels of the expressed TSC2, TSC1, total S6K and T389-phosphorylated S6K were estimated by immunoblotting using the following antibodies: 1A5 anti-Thr^389^ phospho- p70 S6 kinase (S6K) mouse monoclonal, 9B11 anti-myc tag mouse monoclonal, anti-myc tag rabbit polyclonal (Cell Signaling Technology, Danvers, MA, USA), and anti-TSC1 and anti-TSC2 rabbit polyclonal^20^. Secondary antibodies were from Li-Cor Biosciences (Lincoln, NE) and blots were scanned using the Odyssey scanner (Li-Cor Biosciences, Lincoln, NE). Signal intensities were measured and normalized to the signals corresponding to wild-type *TSC2*.

### Predicting pathogenicity

Identified sequence variations were classified into five categories: class 5 (pathogenic), class 4 (likely pathogenic), class 3 (variant of unknown significance), class 2 (likely benign), and class 1 (benign), according to the guidelines of ACMG^21^. Variants were classified as pathogenic based on allelic frequency, and the predicted effect of the variant on *TSC1* or *TSC2*. Variants that occur relatively often in the general population (gnomAD:>1:5000), are unlikely to cause TSC and were classified as benign and only reported if the variant had been previously categorized as pathogenic in the HGMD database. Information obtained from the Leiden Open Variation Database (LOVD) (http://chromium.lovd.nl/LOVD2/TSC/home.php?select_db=TSC1) was used to help variant classification. Rare (gnomAD: <1:5000) variants which led to a frameshift, and/or created a stop codon were classified as pathogenic or likely pathogenic. Determining the pathogenicity of rare missense variants and in-frame duplications or deletions, is often difficult. In addition to allele frequency, these variants were classified according to the results of *in vitro* functional assessment. To investigate possible effects of the identified variants on splicing, we used several web-based tools (MaxEntScan^22^, NNSPLICE^23^, and Human Splice Finder^24^) combined in Alamut Visual biosoftware (http://www.interactive-biosoftware.com/alamut-visual/). Rare variants resulting in a 99–100% reduction in the prediction score were classified as pathogenic. Otherwise we classified the variant as a variant of uncertain clinical significance (VUS).

### Ethics statement

This study is approved by the local institutional review board, Pactius (P-2019-304). No other permission was required. Written informed consent was waived. All methods were carried out in accordance with the Copenhagen University Hospital’s, Rigshospitalets, guidelines.

## Results

### Identification of sequence variants

Molecular testing of *TSC1* and *TSC2* in 327 Danish individuals suspected of TSC resulted in the identification of 137 different variants in a total of 168 individuals. The *TSC1* and *TSC2* variants identified in our cohort are summarized in Supplementary Tables 3 and 4.

The majority of the variants had been reported previously in other TSC cohorts but 45 were novel, as defined by their absence from the HGMD (http://www.hgmd.cf.ac.uk/ac/index.php), LOVD (Leiden Open Variation Database (http://chromium.lovd.nl/LOVD2/TSC/home.php?select_db=TSC1)), and Clin Var (https://www.ncbi.nlm.nih.gov/clinvar/). The 8 novel *TSC1* variants and 37 novel *TSC2* variants are listed in Tables 1 and 2.

**Table 1:** Novel predicted pathogenic variants identified in this study in *TSC1*.

Novel Predicted Pathogenic Variants Identified in this Study in *TSC1*				
Position	Coding effect	Mutation	Annotation	Notes
Exon 7	Deletion	c.554del	p.(Tyr185Serfs*25)	
Exon 15	Nonsense	c.1677C > A	p.(Cys559*)	
Exon 17	Deletion	c. 2065del	p.(Arg689Alafs*35)	
Exon 18	Nonsense	c.2359G > T	p.(Glu787*)	
Exon 19	Deletion	c.2419del	p.(Ile807Leufs*6)	
Exon 19	Deletion	c.2501del	p.(Lys834Serfs*15)	
Intron 21	Splicing	c.2813 + 2T > C	p?	Not present in gnomAD. Predicted change at donor site 2 bps upstream: 100%
**Novel variants of uncertain pathogenicity identified in this study in** ***TSC1***				
Intron 20	Splicing	c.2626-4T > G	p?	Not present in gnomAD. Predicted change at acceptor site 4 bps downstream:−1.4%

**Table 2:** Novel predicted pathogenic variants identified in this study in *TSC2*.

Novel Predicted Pathogenic Variants Identified in this Study In *TSC2*				
Position	Coding effect	Mutation	Annotation	Notes
Exon 2	Duplication	c.62dup	p. (Thr23Asnfs*12)	
Exon 4	Deletion	c.313_337del	p. (LeuAladelfs*69)	
Exon 9	Missense	c.815C > A	p.(Ala272Asp)	Not present in gnomAD
Exon 12	Deletion	c.1120_1130del	p.(Thr347Profs*9)	
Exon 13	Deletion	c.1264delT	p.(Ser422Profs*3)	
Exon 13	Deletion	c.1283_1285del	p. (Ser428del)	Not present in gnomAD. 8% mosaic
Exon 14	Duplication	c.1401_1422dup22bp	p.(Ile475Argfs*14)	
Exon 16	Duplication	c.1699_1701dup	p.(Leu598dup)	Not present in gnomAD
Exon 18	Duplication	c.1875dupA	p.(Leu626Thrfs*31)	
Exon 20	Deletion	c.2176del	p.(Ser726Profs*45)	
Exon 21	Nonsense	c.2285T > A	p.(Leu762*)	
Exon 21	Missense	c.2326T > G	p.(Tyr776Asp)	Not present in gnomAD
Exon 23	Delins	c.2571delins21 (GGCCAGGCTGCCGCACCTCTC)	p.(Tyr857*)	
Exon 27	Deletion	c.3125delC	p.(Pro1042Argfs*11)	
Exon 28	Missense	c.3206T > G	p.(Val1069Ala)	Not present in gnomAD p.(Val1069Glu) Reported as de novo (LOVD)
Exon 29	Deletion	c.3290del	p.(Ser1097Thrfs*6)	
Exon 31	Duplication	c.3682dup	p.(Leu1228Profs*6)	
Exon 31	Deletion	c.3712_3715del	p.(Ala1238Serfs*86)	
Exon 34	Insertion	c.4145_4146insC	p.(Ser1383Glufs*31)	
Exon 34	Delins	c.4315_4326delinsCT	p.(Gly1439Leufs*67)	
Exon 35	Deletion	c.4535_4539del	p.(Asp1512Valfs*10)	
Exon 39	Insertion	c.5059_5060insT	p.(Cys1687Leufs*19)	
Exon 39	Deletion	c.5065_5068 + 1del	p.(Lys1689Thrfs*136)	
Exon 40	Duplication	c. 5116_5119dup	p.(Asn1707Thrfs*23)	
Exon 41	Deletion	c.5212del	p.(Ser1738Profs*88)	
Intron 11	Splicing	c.1120-2A > G	p?	Not present in gnomAD. Predicted effect on splicing: 100%
Intron 12	Splicing	c.1258-2delA	p?	Not present in gnomAD. Predicted effect on splicing: 100%
Intron 13/ Exon 14	Delins	c.1362-63_1382delinsCAG	p?	Not present in gnomAD
Intron 15	Splicing	c.1600-1G > T	p?	Not present in gnomAD. Predicted effect on splicing:100%
Intron 36	Splicing/ deletion	c.4663-27_4668del	p?	Not present in gnomAD. Predicted effect on splicing 100%
Exon 2-10	Deletion	Ex2_10del	c.1-?_975 + ?	
Exon 14	Deletion	Ex14del	c.(1361 + 1_1362-1)_ (1443 + 1_1444-1)del	
Exon 17–29	Deletion	Ex17_29del	c.(1716 + 1_1717-1)_ (3397 + 1_3398-1)del	
**Novel variants of uncertain pathogenicity identified in this study in** ***TSC2***				
Intron 5	Splicing	c.336 + 14C > T	p?	gnomAD frequency All: 0.0040%. Predicted effect on splicing: 0%
Intron 11	Splicing	c.976-16C > A	p?	Not present in glomAD. Predicted effect on splicing: 38.6%
Intron 28	Splicing	c.3284 + 3G > A	p?	Not present in gnomAD. Predicted change at donor site 3 bps upstream: +68.4%
Intron 42	Splicing/deletion.	c.5260-34_5260-10del	p?	Not present in gnomAD. Predicted effect on splicing: 8.9%

Most of the new variants lead to formation of a premature stop codon. This was the case for 20 of the novel *TSC2* variants and for six of the novel *TSC1* variants. Five *TSC2* variants were predicted to lead to an amino acid substitution or an in-frame deletion/insertion.

### Classification of variants

Unlike variants leading to premature termination of translation which can mostly be classified as pathogenic or likely pathogenic, classification of missense and in-frame deletion/insertion variants can be difficult. Functional investigation of several of the identified *TSC1* and *TSC2* variants had been performed previously. The *TSC1* p.(Leu50Pro) variant^15^, and the *TSC2* p.(Arg611Gln)^25^, p.(Phe897Ser)^26^, p.(Arg905Trp)^26^, p.(Arg1032Pro)^15^, p.(Gln1554His)^17^, p.(Arg1570Trp)^17^, p.(Gly1642Asp)^15^, p.(Ser1653Phe)^27^, p.(Pro1675Leu)^27^, p.(Pro1709Leu)^27^, p.(Arg1743Gln)^27^, p.(Arg1743Trp)^28^ and p.(His1746_Arg1751del)^27^ variants have all been found to disrupt TSC complex function (Supplementary Tables 3 and 4).

For the *TSC2* p.(Leu292Pro), p.(Glu1558Lys), p.(His1620Tyr), p.(Lys1638del), p.(Asn1681Lys) and p.(Pro1675Leu) variants, *de novo* occurrence was noted in LOVD, indicating pathogenicity.

To help classify the remaining missense variants and in-frame deletions/insertions identified in our cohort we performed *in vitro* functional assessment. We derived expression constructs for the following *TSC2* variants: c.815C > A, p.(Ala272Asp), c.1283_1285del, p.(Ser428del), c.1699_1701dup, p.(Leu568dup), c.2326T > G, p.(Tyr776Asp), c.1292C > T, p.(Ala431Val) and c.1915C > T, p.(Arg639Trp). The c.1291C > T, p.(Ala431Val) and c.1915C > T, p.(Arg639Trp) variants were inherited in *cis* on the paternal allele. Therefore, we derived an expression construct containing both variants, referred to as p.(Ala431Val/Arg639Trp). Furthermore, expression constructs were generated for the previously identified *TSC2* c.856A > G, p.(Met286Val), c.1220_1240del, p.(Tyr407_Arg413del), c.1853T > C, p.(Leu618Pro), c.4672G > A, p.(Glu1558Lys) and c.5043C > G, p.(Asn1681Lys) variants.

**Figure 2:**
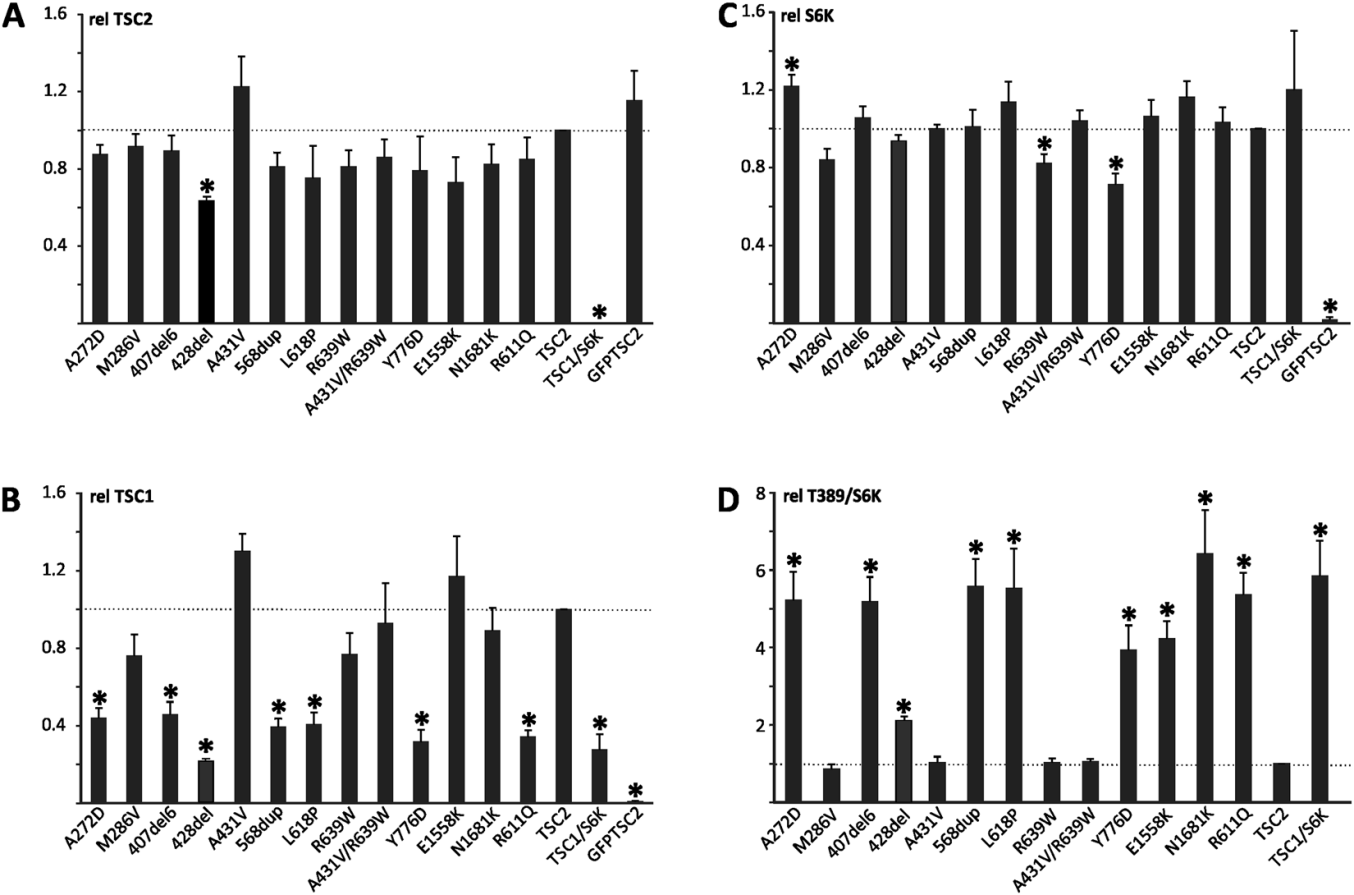
Functional assessment of missense variants, Functional assessment of the *TSC2* (NM_000548.3) variants; c.815C > A, p.(Ala272Asp), c.856A > G, p.(Met286Val), c.1220_1240del, p.(Tyr407_Arg413del), c.1283_1285del, p.(Ser428del), c.1292C > T, p.(Ala431Val), c.1699_1701dup, p.(Leu598dup), c.1853T > C, p.(Leu618Pro), c.1915C > T, p.(Arg639Trp), c.1292C > T/c.1915C > T, p.(Ala431Val/Arg639Trp), c.2326T > G, p.(Tyr776Asp), c.4672G > A, p.(Glu1558Lys) and c.5043C > G, p.(Asn1681Lys). 3H9-1B1 (*TSC2*/*TSC1* double knockout HEK 293 T) cells were transfected with the indicated combinations of expression constructs. Twenty-four hours after transfection the cells were harvested, and the cleared cell lysates analysed by immunoblotting. The signals for TSC2, TSC1, total S6K (S6K) and T389-phosphorylated S6K (T389) were determined per variant, relative to the wild-type control (TSC2) in 2 independent experiments. The mean TSC2 (**A**), TSC1 (**B**) and S6K (**C**) signals and mean T389/S6K ratio (**D**) are shown for each variant. In each case the dotted line indicates the signal/ratio for wild-type TSC2 (=1.0). Error bars represent the standard error of the mean; variants that were significantly different from wild-type TSC2 are indicated with an asterisk (*P* < 0.025; Student’s paired t-test). Amino acid changes are given according to *TSC2* cDNA reference transcript sequence NM_000548.3.

Variants were expressed in 3H9-1B1 (*TSC2*:*TSC1* double knockout HEK 293 T) cells together with *TSC1* and a *S6K* reporter construct. The levels of the exogenous TSC2, TSC1, total S6K and T389-phosphorylated S6K proteins were estimated by immunoblotting. The stability of the expressed TSC2 and the stability of the TSC complex were estimated from the TSC2 (Fig. 2A) and TSC1 (Fig. 2B) signals respectively. The total S6K signal was used to estimate the relative transfection efficiency (Fig. 2C) and the ratio of the signals for T389-phosphorylated S6K and total S6K (T389/S6K ratio) was used to estimate mTORC1 activity (Fig. 2D).

The TSC2 p.(Ala272Asp), p.(Tyr407_Arg413del), p.(Ser428del), p.(Leu568dup), p.(Leu618Pro), p.(Tyr776Asp), p.(Glu1558Lys) and p.(Asn1681Lys) variants disrupted TSC complex function. In each case, mTORC1 activity, as estimated from the T389/S6K ratio, was significantly increased, compared to wild-type TSC2. In addition, the p.(Ala272Asp), p.(Tyr407_Arg413del), p.(Ser428del), p.(Leu568dup), p. (Leu618Pro) and p.(Tyr776Asp) variants were associated with significantly decreased TSC1 signals, most likely due to their inability to interact with and stabilize TSC1. The TSC2 p.(Met286Val), p.(Ala431Val), p.(Arg639Trp) and p.(Ala431Val;Arg639Trp) variants did not significantly disrupt the TSC complex dependent inhibition of mTORC1 activity in our assay, nor did they significantly affect TSC1 or TSC2 signals.

The p.(Met286Val), p.(Ala431Val), and p.(Arg639Trp) variants are all reported in gnomAD with an individual overall frequency of 0.18%, 0.038% and 0.0040% respectively. In comparison, none of the other missense or in-frame variants were present in gnomAD. For the p.(Met286Val) variant, conflicting conclusions regarding pathogenicity were registered. The variant was originally classified as pathogenic or most likely pathogenic in the HGMD database, but as neutral in the LOVD database. The gnomAD frequency for this variant is as high as 1.9% in the East Asian population. Based on the observed activity and stability of the p.(Met286Val) variant in our assay, combined with the gnomAD frequency, we classified the p.(Met286Val), p.(Ala431Val), p.(Arg639Trp) and p.(Ala431Val;Arg639Trp) variants as benign or most likely benign (Table 3).

**Table 3:** Functional investigations of *TSC2* variants.

Functional Investigations Of *TSC2* Variants					
Variants leading to disrupted TSC-complex function					
Position	Coding effect	Mutation	Annotation	Reference Notes	
Exon 9	Missense	c.815C > A	p.(Ala272Asp)	This study	Not present in gnomAD
Exon 12	Deletion	c.1220_1240del21	p.(Tyr407_Arg413del)	17	Not present in gnomAD
Exon 13	Deletion	c.1283_1285del	p.(Ser428del)	This study	Not present in gnomAD 7% mosaic
Exon 16	Duplication	c.1699_1701dup	p.(Leu568dup)	This study	Not present in gnomAD
Exon 18	Missense	c.1853T > C	p.(Leu618Pro)	LOVD	Not present in gnomAD
Exon 21	Missense	c.2326T > G	p.(Tyr776Asp)	This study	Not present in gnomAD
Exon 37	Missense	c.4672G > A	p.(Glu1558Lys)	17	Not present in gnomAD. Reported as de novo (LOVD).
Exon 39	Missense	c.5043C > G	p.(Asn1681Lys)	36	Not present in gnomAD. Reported as de novo (LOVD).
**Variants with no effect on the TSC-complex function**					
Exon 10	Missense Benign	c.856A > G	p.Met286Val	30	gnomAD frequency: All: 0.18%. East Asian: 1.9%
Exon 18	Missense Both Likely benign	c.1915C > T/c.1292C > T	p.Arg639Trp/p.Ala432Val	LOVD	gnomAD frequency. All:0.0041% and 0.038% respectively

Variants located in and around canonical splice sites can be difficult to classify. We identified five novel variants, including one in *TSC1* and four in *TSC2*, that were absent from gnomAD and were predicted to be >99% likely to affect splicing according to web-based tools (MaxEntScan^22^, NNSPLICE^23^, and Human Splice Finder^24^) combined in Alamut Visual biosoftware. We classified these variants as pathogenic or likely pathogenic. Furthermore, we identified the *TSC1* c.2042-5 A > G variant, which was predicted to affect splicing with 34% probability. This variant has been identified previously as a *de novo* change in an individual with TSC (http://chromium.lovd.nl/LOVD2/TSC/home.php?select_db=TSC1). Therefore, we classified the variant as likely to be pathogenic (Supplementary Tables 3 and 4).

In addition, we identified the novel *TSC1* c.2626-4 T > G and *TSC2* c.976-16 C > A, c.3284 + 3 G > A and c.5260-34_5260-10del variants, as well as the previously identified *TSC2* c.3883 + 5 C > T variant. These variants are all predicted to affect splicing but at a probability significantly below 100% (between 1% and 68%). We classified all these variants as VUS. We classified also the novel *TSC2* c.336 + 14 C > T variant, predicted to have no effect on splicing, as VUS (Supplementary Tables 3 and 4). Unfortunately it was not possible to investigate the effects of the variants on *TSC1* and *TSC2* pre-mRNA splicing in the corresponding affected individuals because no RNA was available from these individuals^11^.

In summary, seven novel predicted pathogenic variants were identified in *TSC1* (Table 1) and 33 in *TSC2* (Table 2). Furthermore, five variants predicted to be of uncertain pathogenicity were identified.

## Discussion

We have reviewed the *TSC1* and *TSC2* variants identified in a cohort of Danish patients, we identified 137 different mutations in 168 TSC patients from a cohort of 327 Danish individuals suspected of TSC. In our cohort, 33 of the 137 different suspected pathogenic variants were identified in *TSC1* (24%) while 104 were identified in *TSC2* (76%) (Table 1). This distribution is in accordance with previous publications^8,29,30^.

In total, 33 different predicted pathogenic *TSC1* variants were identified in 42 individuals and 106 different predicted pathogenic *TSC2* variants were identified in 126 individuals. In addition to the predicted pathogenic variants, 8 variants with uncertain pathogenicity were identified (Supplementary Tables 3 and 4). Twenty variants, 7 in *TSC1* and 13 in *TSC2*, were identified more than once in our cohort. The most common variants in *TSC1* were the nonsense mutations c.1525C > T, p.(Arg509*) in exon 15 and c.2074C > T, p.(Arg692*) in exon 17, both identified in three unrelated patients. In *TSC2*, c.1832G > A, p.(Arg611Gln) located in exon 17 and c.5238_5255del, p.(His1746_Arg1751del) in exon 41 were the most common variants, identified in 5 unrelated patients each. In six cases molecular testing was performed on individuals in whom cardiac rhabdomyomas had been revealed by prenatal ultrasound scanning. Pathogenic variants were identified in five of these cases: *TSC2* c.1832G > A, p.(Arg611Gln), c.4537G > T, p.(Glu1513*), c.4993C > T, p.(Gln1665*), c.5024C > T, p.(Pro1675Leu), and c.(1-?)_(975 + ?)del (del Ex2-10).

All the predicted pathogenic variants identified in *TSC1* were small changes, involving a single base pair (30 cases), two base pairs (2 cases) or 23 base pairs (1 case). In 25 cases, the identified change created a premature stop codon, and in seven cases, the variant was in a region important for splicing. Only a single variant predicted to lead to an amino acid substitution was identified. In *TSC2*, 73 variants affected a single base-pair and 22 variants affected between two and 33 base-pairs. In 54 of these cases a premature stop codon was created, in 15 cases the variant was in a region important for splicing and in 24 cases the variant was predicted to change the amino acid sequence. Furthermore, 9 variants leading to large deletions of one or more exons of *TSC2* were identified.

Most of the identified variants had been identified previously in other TSC patients, but a total of 33 novel predicted pathogenic variants were identified in *TSC2* and 7 novel predicted pathogenic variants were identified in *TSC1*.

**Figure 3:**
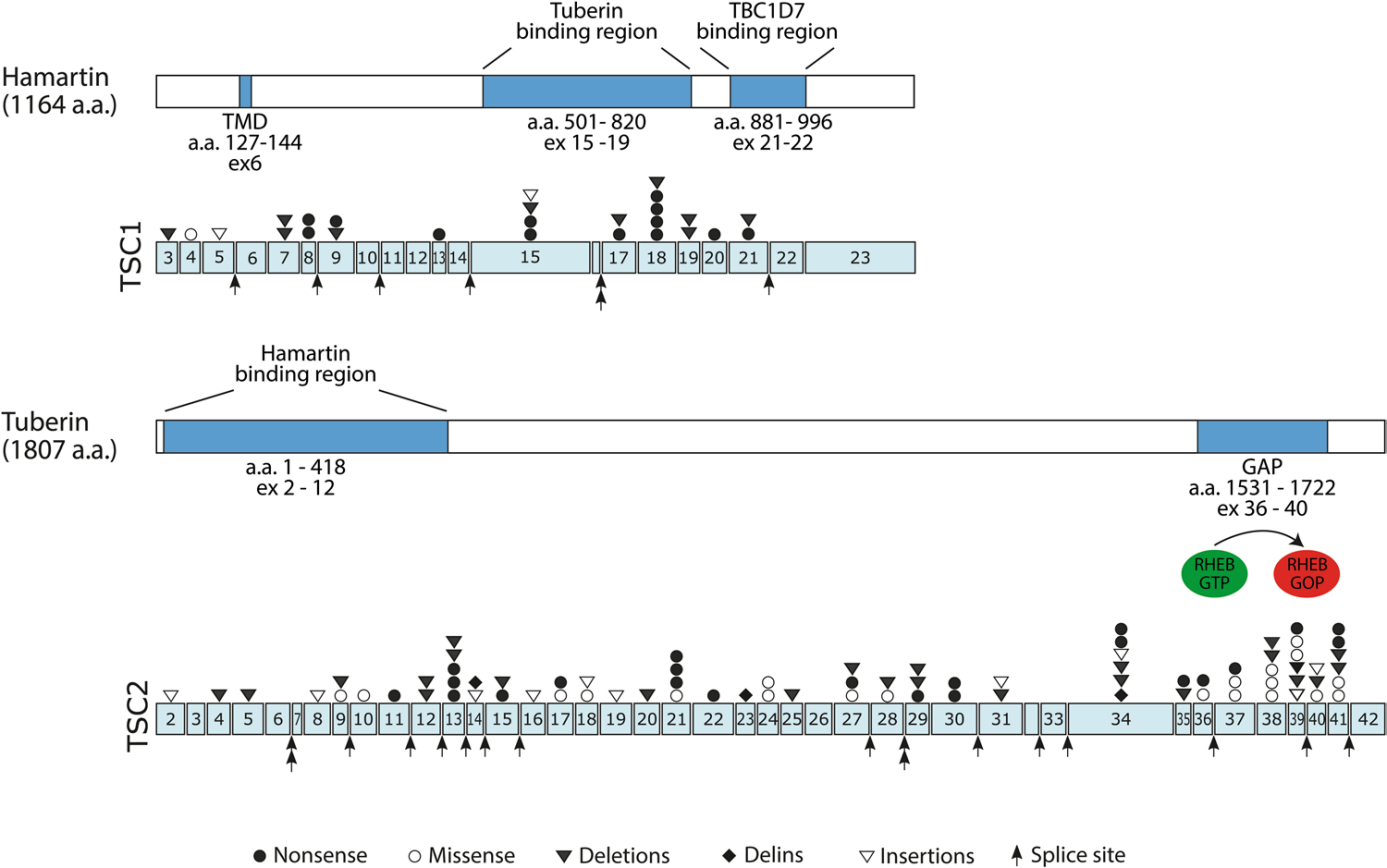
Structural and functional domains of TSC1 and TSC2 and the distribution of the *TSC1* and *TSC2* variants identified in this study. TSC1 consists of a conserved N-terminal domain (NTD) and a large domain that is critical for the interaction with the N-terminal of TSC2, and a domain at the C-terminus important for interacting with the TBC1D7. TSC2 is the catalytic subunit of the TSC complex and acts as a GTPase activating protein towards RHEB. The active site is indicated. The types and frequencies of the *TSC1* and *TSC2* variants identified in in our cohort are illustrated. In total, 33 different pathogenic variants in TSC1 and 104 different pathogenic variants in TSC2 were identified in 168 patients. The variants were categorized as either missense, nonsense, splicing, small deletions, small insertions, or small delins. Gross deletions (9 in TSC2) are not shown. The figure is modified from previous publications^34–36^.

The observed distributions of pathogenic *TSC1* and *TSC2* variants, shown in Fig. 3, are similar to previous studies^8,29,30^. *TSC2* variants were scattered all over the gene and *TSC1* variants were most often identified in exons 15 and 18. Although most of the variants were either nonsense changes or deletions, missense mutations were often found in *TSC2*. In contrast, only a single missense variant was identified in *TSC1*.

Missense and small in-frame indel variants encode proteins that only differ from the wild-type proteins by a few amino acids. If a single amino acid substitution is pathogenic, then it is likely that that amino acid and/or the surrounding peptide sequence is functionally important. The only missense variant in *TSC1* identified in this study, p.(Leu50Pro), affects the N-terminal domain (NTD) of TSC1, resulting in destabilization of the TSC complex^31^. A high proportion, (11/19; ~60%) of the *TSC2* missense variants identified map to exons 36–41, encoding amino acids 1555–1704, that contain the GAP domain (amino acids 1533–1722), even though this region only accounts for ~11% of the total coding region. Furthermore, two pathogenic missense variants affecting this region were identified in multiple cases. These results indicate that the NTD region of TSC1 and the GAP domain of TSC2 are critical for TSC complex function. Our functional analysis of the p.(Glu1558Lys) and p.(Asn1681Lys) variants is in line with this hypothesis.

Previous studies report a mutation detection rate of 74–83% in TSC^8,29,30,32^. In the present study we identified a mutation in only 52% of the patients. This is in contrast to a previous publication from our laboratory^17^, where 65 Danish patients who had been clinically diagnosed with TSC, were investigated and pathogenic mutations were identified in 51 patients (78%). In the present study only limited clinical information was available, whereas all the patients included in our previous study fulfilled the diagnostic criteria for TSC. At that time NGS was still not available and *TSC1* and *TSC2* molecular screening was difficult and time consuming. This might have forced the clinician to carefully evaluate their patient for signs of TSC prior to referral for molecular genetic investigation. Today, with NGS, the laboratory work is reduced, and the turn-around time faster. This might lead to increased numbers of patients being referred who do not fulfill the clinical diagnostic criteria for TSC. The large number of cases without identification of a pathogenic *TSC1* or *TSC2* variant does not exclude the possibility that these individuals have TSC. The variant could be located in a region not tested in any of our set ups, like deep within an intron, or the variants might be present in mosaic form, in a limited number of patient cells. Indeed, recent studies indicate that at least 50% of TSC cases who fulfill the clinical diagnostic criteria and do not have a mutation identified by standard molecular testing will have a pathogenic *TSC1* or *TSC2* variant in mosaic form^12,13^. Only a minor fraction of the cases presented here were screened using NGS. So far, we have identified mosaicism in one case. The *TSC2* c.1283_1285del variant was identified in 84 out of 1082 reads (8%) and was verified by PCR using deletion specific primers. The further application of NGS should lead to an increase in the number of clarified cases. Also, re-investigation of mutation-negative cases might reveal additional pathogenic variants in mosaic form.

Careful re-assessment of all the previously published mutations identified in our cohort revealed conflicting interpretations of pathogenicity. The release of the genome aggregation database (gnomAD) which is comprised of data from 123,136 individuals and whole genome sequencing from 15,496 individuals^33^ has increased our knowledge about the frequencies of many single nucleotide variants (SNPs), and led us to re-classify some variants as unlikely to be disease causing. Furthermore, assessment of pathogenicity using functional studies helped support the genetic and clinical data. For example, re-classification of the *TSC2*, p.(Met286Val) variant as benign was supported by both the frequency data and the functional assessment.

Reliable classification of identified variants is critically important. Functional *in vitro* investigation is an important contribution to classification of variants leading to missense changes and in frame deletions and insertions. Routine investigation of potential splice-site mutations by reverse-transcription (RT)-PCR performed on RNA isolated from the affected individuals might also help improve the classification of variants, particularly those located in splice site regions.

## Supplementary information

Supplementary Tables 1, 2, 3, 4.
