# Supplementary material for: Mutational analysis of TSC1 and TSC2 in Danish patients with tuberous sclerosis complex
Source: Sci Rep. 2020 Jun 18;10:9909. doi: 10.1038/s41598-020-66588-4 (PMC7303179; doi:10.1038/s41598-020-66588-4)
Supplement: Supplementary file 1 — Supplementary Tables 1, 2, 3, 4. [file 41598_2020_66588_MOESM1_ESM.pdf]

Supplementary files

Mutational analysis of *TSC1* and *TSC2* in Danish patients with tuberous sclerosis complex

Thomas Rosengren, Santoesha Nanhoe, Luis Gustavo Dufner de Almeida, Bitten Schönewolf-Greulich, Lasse Jonsgaard Larsen, Caroline Amalie Brunbjerg Hey, Morten Dunø, Jakob Ek, Lotte Risom, Mark Nellist, Lisbeth Birk Møller

Exon	Primer<br>(L:left, R:right)	Sequence<br>Lower letters: universal tags, upper letters: gene specific
3	ABI-TSC1_Ex3F	acccactgcttactggcttatcATTGCATTGAACACGTGCAT
	ABI-TSC1_Ex3R	gaggggcaaacaacagatggcCAGCAGGATTCTAGTGGCTCT
4	ABI-TSC1_Ex4F	acccactgcttactggcttatcCATATTTAGGCAAATAAGCTTGG
	ABI-TSC1_Ex4R	gaggggcaaacaacagatggcTTTTCAAGAATCATGGGTCC
5	ABI-TSC1_Ex5F	acccactgcttactggcttatcTTGAGAGATTGGAGCACATCA
	ABI-TSC1_Ex5R	gaggggcaaacaacagatggcAGCTTCCTTGCTTTAAGTTGC
6	ABI-TSC1_Ex6F	acccactgcttactggcttatcTCCTTAGGGATAGGGAAATGA
	ABI-TSC1_Ex6R	gaggggcaaacaacagatggcTGGATGCACCCAAGATATTC
7	ABI-TSC1_Ex7F	acccactgcttactggcttatcTTCGGATTTGTTTGCATTTT
	ABI-TSC1_Ex7R	gaggggcaaacaacagatggcGCCTAGGGATTGGAGTGG
8	ABI-TSC1_Ex8F	acccactgcttactggcttatcATCCCTAGGCAGCCACTAA
	ABI-TSC1_Ex8R	gaggggcaaacaacagatggcTGAGGTGTAAGCCTTTCTGG
9	ABI-TSC1_Ex9F	acccactgcttactggcttatcGGCTGGCACTGAGTTGACAC
	ABI-TSC1_Ex9R	gaggggcaaacaacagatggcGGGAAAAATCCCTAGGAACTGA
10	ABI-TSC1_Ex10F	acccactgcttactggcttatcGACTTCAAGATGAATCTAAGAGGC
	ABI-TSC1_Ex10R	gaggggcaaacaacagatggcCCTAAAACCACACACTAACCCC
11	ABI-TSC1_Ex11F	acccactgcttactggcttatcGCAACTTGTTTCTTGATACGC
	ABI-TSC1_Ex11R	gaggggcaaacaacagatggcCACATTCTGAAAGCCCCA
12	ABI-TSC1_Ex12F	acccactgcttactggcttatcGATCCCCAGAAAGTTAACTCTAGC
	ABI-TSC1_Ex12R	gaggggcaaacaacagatggcTGAGAGCAGCTTGTTAGTCCA
13	ABI-TSC1_Ex13F	acccactgcttactggcttatcCATCCCAACAATTTGAGAATCA
	ABI-TSC1_Ex13R	gaggggcaaacaacagatggcTGTTTAGGCCTCAGTATTTTGGA
14	ABI-TSC1_Ex14F	acccactgcttactggcttatcCCATGTCCAGCCTTCTCTGT
	ABI-TSC1_Ex14R	gaggggcaaacaacagatggcTACCTGGCATAGGTCCCAGA
15a	ABI-TSC1_Ex15aF	acccactgcttactggcttatcTGAGTGACACTGGCATGTGG
	ABI-TSC1_Ex15aR	gaggggcaaacaacagatggcGCAATGCCACCTCAAAAAGA
15b	ABI-TSC1_Ex15bF	acccactgcttactggcttatcTCGAGGAGGCTTTGACTCTC
	ABI-TSC1_Ex15bR	gaggggcaaacaacagatggcATGCAACAGCCTAGAAGGACA
16	ABI-TSC1_Ex16F	acccactgcttactggcttatcCTGTGGACCTGGAGTTTGAA
	ABI-TSC1_Ex16R	GaggggcaaacaacagatggcTGACTTGGCAACACTTGAGA
17	ABI-TSC1_Ex17F	acccactgcttactggcttatcCTGGGAGTCAAGTGGTCATT
	ABI-TSC1_Ex17R	gaggggcaaacaacagatggcCTTAATCTCAAGCGACCTGC
18	ABI-TSC1_Ex18F	acccactgcttactggcttatcTGACCTAAGGCCAAGTAAGACA
	ABI-TSC1_Ex18R	gaggggcaaacaacagatggcGCTGAACAAGTCAAGGACACC
19	ABI-TSC1_Ex19F	acccactgcttactggcttatcAGGGCCCAAGAAAGTAGAGC
	ABI-TSC1_Ex19R	gaggggcaaacaacagatggcATTGCCAAATGTCAGGGACT
20	ABI-TSC1_Ex20F	acccactgcttactggcttatcTTGTCCTTGGGTACCATCCT

	ABI-TSC1_Ex20R	gaggggcaaacaacagatggcACCACGGAGTAGTGGGACTG
21	ABI-TSC1_Ex21F	acccactgcttactggcttatcTGCCTTCTCAGTCCTTCTTACAT
	ABI-TSC1_Ex21R	gaggggcaaacaacagatggcCCTTTTCTGAGCCTCATACCT
22	ABI-TSC1_Ex22F	acccactgcttactggcttatcGCCAAGAAAGACCACCTTC
	ABI-TSC1_Ex22R	gaggggcaaacaacagatggcTTTGAGGAAAGGAACGTCAG
23	ABI-TSC1_Ex23aF	acccactgcttactggcttatcGGCTAGCGGAGTTCAGTGTC
	ABI-TSC1_Ex23aR	gaggggcaaacaacagatggcAGCCTTCATACCCAGGAAGC
24	ABI-TSC1_Ex23bF	acccactgcttactggcttatcGGGCAGTAGTGGAAGCAGAG
	ABI-TSC1_Ex23bR	gaggggcaaacaacagatggcCAATGCCAGATCCAAAAACC

Supplementary Table 1. Primers used for Sanger sequencing of TSC1.The primers are all extended with the universal forward primer: acccactgcttactggcttatc or the universal reverse primer: gaggggcaaacaacagatggc. Sanger sequencing of the PCR products are performed using the universal primers.

Exon	Primer<br>(L:left, R:right)	Sequence<br>Lower letters: universal tags, upper letters: gene specific
UTR	ABI-TSC2-UTRL	acccactgcttactggcttatcGCGGCACAGAACTACAACTC
	ABI-TSC2-UTRR	gaggggcaaacaacagatggcAGTCGGGTTGCAGTGGG
1	ABI-TSC2-ex1L	acccactgcttactggcttatcTGTGGGAGGAAAGGTTATGC
	ABI-TSC2-ex1R	gaggggcaaacaacagatggcCCTGTGTAGACAGTTGCCAGC
2	ABI-TSC2-ex2L	acccactgcttactggcttatcAGTCTGGAAAATGCAGTGGG
	ABI-TSC2-ex2R	gaggggcaaacaacagatggcAAACCAGATCATCGGCAGTC
3	ABI-TSC2-ex3L	acccactgcttactggcttatcGTTGTTCCTCCCTGTCCTCC
	ABI-TSC2-ex3R	gaggggcaaacaacagatggcATCAGCCCCTAGACCCTGTG
4	ABI-TSC2-ex4L	acccactgcttactggcttatcGGAAGGAGAGGGGTCCAG
	ABI-TSC2-ex4R	gaggggcaaacaacagatggcCATCCCTGAGAACGGCAG
5	ABI-TSC2-ex5L	acccactgcttactggcttatcACTGATGATGGGGTTTCTGG
	ABI-TSC2-ex5R	gaggggcaaacaacagatggcATTGTGCCCAGCCCAAC
6	ABI-TSC2-ex6L	acccactgcttactggcttatcGCCATGCGTGTTATTGACG
	ABI-TSC2-ex6R	gaggggcaaacaacagatggcCTCAGAGAGACCGAGCAGC
7	ABI-TSC2-ex7L	acccactgcttactggcttatcAGCGCAGGCTGAAGGAG
	ABI-TSC2-ex7R	gaggggcaaacaacagatggcGCCACCCCAAGAATCAGAC
8	ABI-TSC2-ex8L	acccactgcttactggcttatcCTATAGGGCAGCAGCCAGG
	ABI-TSC2-ex8R	gaggggcaaacaacagatggcCTTGGCAAGGGACACTGG
9	ABI-TSC2-ex9L	acccactgcttactggcttatcCACAGGGACCTCTGGGG
	ABI-TSC2-ex9R	gaggggcaaacaacagatggcAAAGGCCTAGAAATGCCACC
10	ABI-TSC2-ex10L	acccactgcttactggcttatcCGCTCAGGCGTGCTACTC
	ABI-TSC2-ex10R	gaggggcaaacaacagatggcAACACGGTTCTGGCAGTCTC
11	ABI-TSC2-ex11L	acccactgcttactggcttatcGGTGGGTGTGTAGCGAGG
	ABI-TSC2-ex11R	gaggggcaaacaacagatggcTCAGAAAGCTGCACTTCACC
12	ABI-TSC2-ex12L	acccactgcttactggcttatcGGGCTCTGACAGCAAACC
	ABI-TSC2-ex12R	gaggggcaaacaacagatggcGCTCTCCAGAGGCAGAGG
13	ABI-TSC2-ex13L	acccactgcttactggcttatcGGAGGACCCAGAGTCGG
	ABI-TSC2-ex13R	gaggggcaaacaacagatggcACCGAAGTCCCAGGCAG
14	ABI-TSC2-ex14L	acccactgcttactggcttatcAGCTGTGCTGAAGTCCCG
	ABI-TSC2-ex14R	gaggggcaaacaacagatggcATCCGGTCACTCGAAGAGG
15	ABI-TSC2-ex15L	acccactgcttactggcttatcGAGCTGAGATTGTGCCACC
	ABI-TSC2-ex15R	gaggggcaaacaacagatggcAAGAGCAGGAGGAAGGTTCTG
16	ABI-TSC2-ex16L	acccactgcttactggcttatcGTGTTTTGAAGCACGCACTC
	ABI-TSC2-ex16R	gaggggcaaacaacagatggcCTGAGGCCCCATCCTGG
17	ABI-TSC2-ex17L	acccactgcttactggcttatcTTTTCTGAGTGCCTGTGGTG
	ABI-TSC2-ex17R	gaggggcaaacaacagatggcGAACGGAACAGACTTGGCTC
18	ABI-TSC2-ex18L	acccactgcttactggcttatcGCTGTTTGCATGTCTGAGGG
	ABI-TSC2-ex18R	gaggggcaaacaacagatggcCACAGGCCAGACAGGGAG
19	ABI-TSC2-ex19L	acccactgcttactggcttatcCATAGCCCTTGACGCTGTG
	ABI-TSC2-ex19R	gaggggcaaacaacagatggcAGTTTGAGGGAAATGGAGCC
20	ABI-TSC2-ex20L	acccactgcttactggcttatcCAAGAAGGCTCCCCAGC
	ABI-TSC2-ex20R	gaggggcaaacaacagatggcGGCCTGGCAGAAACTCG
21	ABI-TSC2-ex21L	acccactgcttactggcttatcCTAAGCCTCGGCTGTTCTCC
	ABI-TSC2-ex21R	gaggggcaaacaacagatggcGAGACACCCAGGTTCCCC
22	ABI-TSC2-ex22L	acccactgcttactggcttatcCTCTGCAGCACCCCATC
	ABI-TSC2-ex22R	gaggggcaaacaacagatggcCCCCAGGGCATGAAGTG
23	ABI-TSC2-ex23L	acccactgcttactggcttatcGCAGCCTTTGTCCCCAAG

	ABI-TSC2-ex23R	gaggggcaaacaacagatggcCCAAACACCCTCCCACTG
24	ABI-TSC2-ex24L	acccactgcttactggcttatcCTAGCCTGCAGCTTGTCCC
	ABI-TSC2-ex24R	gaggggcaaacaacagatggcCAGGACCCATTTCCACTCAC
25	ABI-TSC2-ex25L	acccactgcttactggcttatcGGGATCTCTCCATCCTGACC
	ABI-TSC2-ex25R	gaggggcaaacaacagatggcGACACGGGCAGACGATG
26	ABI-TSC2-ex26L	acccactgcttactggcttatcCTGTTGGGGTCTTTCCGAG
	ABI-TSC2-ex26R	gaggggcaaacaacagatggcCTAGGAGGAACTCGCCCAC
27	ABI-TSC2-ex27L	acccactgcttactggcttatcGTCTTCTCCAACTTCACGGC
	ABI-TSC2-ex27R	gaggggcaaacaacagatggcCACGCACAGGGTGGACTTAG
28	ABI-TSC2-ex28L	acccactgcttactggcttatcCTGCCAGCCTCGACACC
	ABI-TSC2-ex28R	gaggggcaaacaacagatggcCTGAAGCCAGCCACCCC
29	ABI-TSC2-ex29L	acccactgcttactggcttatcGTCTCTGGCTGCCTGTGG
	ABI-TSC2-ex29R	gaggggcaaacaacagatggcCCCCAAATATCCCAAGAGG
30	ABI-TSC2-ex30L	acccactgcttactggcttatcGGAGCATGAGGGCAAAAC
	ABI-TSC2-ex30R	gaggggcaaacaacagatggcCAGGCCAGGGACCACTC
31	ABI-TSC2-ex31L	acccactgcttactggcttatcCCTGCCCTCTCTCCTCTG
	ABI-TSC2-ex31R	gaggggcaaacaacagatggcAATGGAGGCAGACGGACC
32	ABI-TSC2-ex32L	acccactgcttactggcttatcGCTGCTGTCCCTCTGGTC
	ABI-TSC2-ex32R	gaggggcaaacaacagatggcCAAGTTCAGAGCCAGTTCCC
33	ABI-TSC2-ex33aL	acccactgcttactggcttatcCCTGGGATGGAGGACAGATAG
	ABI-TSC2-ex33aR	gaggggcaaacaacagatggcCGACCAGGCAGCACTTTC
	ABI-TSC2-ex33bL	acccactgcttactggcttatcGGACATCCTCGGGGACC
	ABI-TSC2-ex33bR	gaggggcaaacaacagatggcCTAGGGCACAGGCGAGG
34	ABI-TSC2-ex34L	acccactgcttactggcttatcGGTGGAGTGGGAGATGGC
	ABI-TSC2-ex34R	gaggggcaaacaacagatggcCTGCCACAGGGAGCTTAGG
35	ABI-TSC2-ex35L	acccactgcttactggcttatcCTGGCCTAAGCTCCCTGTG
	ABI-TSC2-ex35R	gaggggcaaacaacagatggcACTTCATGCTGTAGGGGACC
36	ABI-TSC2-ex36L	acccactgcttactggcttatcCCTCAGGGATCAGAGTGGG
	ABI-TSC2-ex36R	gaggggcaaacaacagatggcGAGCTGGAGCAGGTGGG
37	ABI-TSC2-ex37L	acccactgcttactggcttatcACCAGAGGACGTGGTCCC
	ABI-TSC2-ex37R	gaggggcaaacaacagatggcTCCTCGTGACCGAAGCTC
38-39	ABI-TSC2-ex38-39L	acccactgcttactggcttatcCTGCCCATGGAGCTGAC
	ABI-TSC2-ex38-39R	gaggggcaaacaacagatggcCTATGATGCACCTGTGAGGC
40	ABI-TSC2-ex40L	acccactgcttactggcttatcCAACCTGCCCTTCGTGG
	ABI-TSC2-ex40R	gaggggcaaacaacagatggcGGCAGTAAGTCTGGGAGGC
41	ABI-TSC2-ex41L	acccactgcttactggcttatcGAATATGGGGCTCCCTCAG
	ABI-TSC2-ex41R	gaggggcaaacaacagatggcCGCACCAAGCAGACAAAGTC

Supplementary Table 2. Primeres used for Sanger sequencing of TSC2. The primers are all extended with the universal forward primer: acccactgcttactggcttatc or the universal reverse primer: gaggggcaaacaacagatggc. Sanger sequencing of the PCR products are performed using the universal primers.

VARIANTS IN TSC1 PREDICTED PATHOGENIC OR LIKELY PATHOGENIC					
**Position**	**Coding effect**	**Mutation**	**Annotation**	**Reference***	**Notes**
Exon 3	Deletion	c.70del	p.(Asp24Thrfs*2)	LOVD[1]	
Exon 4	Missense	✦c.149T>C	p.(Leu50Pro)	LOVD	Not present in gnomAD. Disrupts function [2]
Exon 5	Duplication	c.252dup	p.(Thr85Hisfs*22)	LOVD[1]	
Exon 7	Deletion	c.563_564del	p.(Phe188Serfs*29)	LOVD[1]	
Exon 7	Deletion	c.554del	p.(Tyr185Serfs*25)	**This study**	
Exon 8	Nonsense	✦c.682C>T	p.(Arg228*)	LOVD[1]	
Exon 8	Nonsense	c.733C>T	p.(Arg245*)	LOVD	
Exon 9	Deletion	c.738del	p.(Arg246Serfs*5)	LOVD[1]	
Exon 9	Nonsense	c.749T>G	**p.(Leu250*)**	LOVD[3]	
Exon 13	Nonsense	c.1303C>T	p.(Gln435*)	LOVD	
Exon 15	Nonsense	✦✦ c.1525C>T	p.(Arg509*)	LOVD[1]	
Exon 15	Deletion	c.1580_1581del	p.(Gln527Argfs*7)	LOVD	
Exon 15	Nonsense	c.1677C>A	p.(Cys559*)	**This study**	
Exon 15	Duplication	c.1680_1702dup	p.(Gly568Alafs*69)	LOVD[1]	
Exon 17	Deletion	c. 2065del	p.(Arg689Alafs*35)	**This study**	
Exon 17	Nonsense	✦✦c.2074C>T	p.(Arg692*)	LOVD[1]	
Exon 18	Nonsense	c.2227C>T	p.(Gln743*)	LOVD	
Exon 18	Nonsense	c.2263C>T	p.(Gln755*)	LOVD	
Exon 18	Nonsense	c.2359G>T	p.(Glu787*)	**This study**	

Exon 18	Nonsense	✦c.2347C>T	p.(Gln783*)	LOVD	
Exon 18	Deletion	c.2364del	p.(Glu788Aspfs*19)	LOVD[1]	
Exon 19	Deletion	c.2419del	p.(Ile807Leufs*6)	**This study**	
Exon 19	Deletion	c.2501del	p.(Lys834Serfs*15)	**This study**	
Exon 20	Nonsense	c.2593C>T	p.(Gln865*)	LOVD	
Exon 21	Deletion	c.2672del	p.(Asn891Thrfs*40)	LOVD	
Exon 21	Nonsense	c.2773G>T	p.(Glu925*)	LOVD	
Intron 5	Splicing	✦c.363+1G>A	p?	LOVD	Not present in gnomAD<br>Predicted change at donor site 1 bps upstream: -100.0%
Intron 8	Splicing	c.737+1G>T	p?	LOVD	Not present in gnomAD. Predicted change at donor site 1 bps upstream: -100%
Intron 10	Splicing	c.1029+1G>A	p?	LOVD	Frequency in gnomAD: 0.00040%<br>Predicted change at donor site 1 bps upstream: -100.0%
Intron 14	Splicing	✦c.1439-1G>A	p?	LOVD	Not present in gnomAD. Predicted change at acceptot site 1bps downstream -100%
Intron 16	Splicing	c.2042-2A>G	p?	LOVD[1]	Not present in gnomAD. Predicted change at acceptor site 2 bps downstream: -100.0%
Intron 16	Splicing	c.2042-5A>G	p?	LOVD[1]	De novo (LOVD)<br>Not present in gnomAD. Predicted change at acceptor site 5 bps downstream: -33.8%
Intron 21	Splicing	c.2813+2T>C	p?	**This study**	Not present in gnomAD Predicted change at donor site 2pbs upstream: -100%
**VARIANT IN TSC1 OF UNCERTAIN PATHOGENIC**					
**Position**	**Coding effect**	**Mutation**	**Annotation**	**Reference**	**Notes**
Intron 20	Splicing	c.2626-4T>G	p?	**This study**	Not present in gnomAD. Predicted change at acceptor site 4 bps downstream: -1.4%

Supplementary Table 3. ***TSC21* variants identified in Danish TSC patients during the period 2003-2018**. Overview of predicted pathogenic or likely pathogenic *TSC1* variants. The lower part shows a selected variant with uncertain pathogenicity (VUS).In silico prediction of novel mutations in TSC1 performed using Alamut including access to MaxEntScan[4], NNSPLICE[5], and Human Splice Finder[6] .

✦ Observed in two unrelated patients; ✦✦ Observed in three unrelated patients

VARIANTS IN TSC2 PREDICTED PATHOGENIC OR LIKELY PATHOGENIC					
**Position**	**Coding effect**	**Mutation**	**Annotation**	**Reference**	**Notes**
Exon 2	Duplication	c.62dup	p.(Thr23Asnfs*12)	**This study**	
Exon 4	Deletion	c.313_337del	p. (LeuAladelfs*69)	**This study**	
Exon 5	Deletion	c. 357del	p.(Arg120Glufs*62)	LOVD[7]	
Exon 8	Duplication	c.710dupC	p.(Leu238Alafs*100)	**ClinVar**	
Exon 9	Missense	c.815C>A	p.(Ala272Asp)	**This study**	Not present in gnomAD.<br>**FA:Disrupts function (this study).**
Exon 9	Deletion	c.826_827del	p. (Met276Valfs*61)	[1]	
Exon10	Missense	c.875T>C	p.(Leu292Pro)	LOVD	Not present in gnomAD.<br>Reported as *de novo* (LOVD).
Exon 11	Nonsense	c.1111C>T	p.(Gln371*)	[1]	
Exon 12	Deletion	c.1120_1130del	p.(Thr347Profs*9)	**This study**	
Exon 12	Deletion	c.1220_1240del21	p.(Tyr407_Arg413del)	[1]	Not present in gnomAD**.**<br>**FA: Disrupts function (this study).**
Exon 13	Deletion	c.1264delT	p.(Ser422Profs*3)	**This study**	
Exon 13	Deletion	c.1283_1285del	p.(Ser428del)	**This study**	Not present in gnomAD.<br>**FA: Disrupts function (this study).**<br>8 % mosaic
Exon 13	Nonsense	c.1287T>G	p.(Tyr429*)	[8]	
Exon 13	Nonsense	c.1294C>T	p.(Gln432*)	LOVD	
Exon 13	Nonsense	c.1322G>A	p.(Trp441*)	LOVD	
Exon 14	Duplication	c.1401_1422dup22bp	p.(Ile475Argfs*14)	**This study**	
Exon 15	Nonsense	✦✦c.1513C>T	p.(Arg505*)	[1,9]	
Exon 15	Deletion	c.1565_1566del	p.(His522Profs*66)	ClinVar	
Exon 16	Duplication	c.1699_1701dup	p.(Leu568dup)	**This study**	Not present in gnomAD<br>**FA: Disrupts function (this study).**
Exon 17	Nonsense	c.1783C>T	p.(Gln595*)	[1,10]	
Exon 17	Missense	✦✦✦✦c.1832G>A	p.(Arg611Gln)	[1,11]	Not present in gnomAD.<br>FA: Disrupts function (LOVD) [12]
Exon 18	Missense	c.1853T>C	p.(Leu618Pro)	LOVD	Not present in gnomAD.

					**FA: Disrupts function (this study)**
Exon 18	Duplication	c.1875dupA	p.(Leu626Thrfs*31)	**This study**	
Exon 19	Insertion	c.2090dupT	p.(Leu697Phefs*6)	[1]	
Exon 20	Deletion	c.2176del	p.(Ser726Profs*45)	**This study**	
Exon 21	Nonsense	✦c.2251C>T	p.(Arg751*)	[1]	
Exon 21	Nonsense	✦c.2353C>T	p.(Gln785*)	[13]	
Exon 21	Nonsense	c.2285T>A	p.(Leu762*)	**This study**	
Exon 21	Missense	c.2326T>G	p.(Tyr776Asp)	**This study**	Not present in gnomAD.<br>**FA: Disrupts function (this study)**
Exon 22	Nonsense	c.2359G>T	p.(Glu787*)	LOVD	
Exon 23	Delins	c.2571delins21<br>(GGCCAGGCTGCCGCACCTCTC)	p.(Tyr857*)	**This study**	
Exon 24	Missense	c.2690T>C	p.(Phe897Ser)	[1]	Not present in gnomAD.<br>FA: Disrupts function (LOVD) [14]
Exon 24	Missense	c.2713C>T	p.(Arg905Trp)	[15]	Not present in gnomAD.<br>FA:Disrupts function(LOVD) [14]
Exon 25	Deletion	✦c.2785del	p.(Glu929Argfs*19)	[1]	
Exon 27	Nonsense	c.3094C>T]	p.(Arg1032*)	ClinVar	
Exon 27	Missense	c.3095G>C	p.(Arg1032Pro)	[16]	Not present in gnomAD<br>FA: Disrupts function (LOVD[17]
Exon 27	Deletion	c.3125delC	p.(Pro1042Argfs*11)	**This study**	
Exon 28	Missense	c.3206T>G	p.(Val1069Ala)	**This study**	Not present in gnomAD<br>p.(Val1069Glu) reported as de novo (LOVD)
Exon 28	Deletion	c.3206_3207del	p.(Val1069Aspfs*98)	[18]	
Exon 29	Deletion	c.3290del	p.(Ser1097Thrfs*6)	**This study**	
Exon 29	Nonsense	✦c.3310C>T	p.(Gln1104*)	LOVD	
Exon 29	Deletion	c.3350_3360del	p.(Gly1117Valfs*47)	[1]	
Exon 30	Nonsense	c.3412C>T	p.(Arg1138*)	[1,10]	
Exon 30	Nonsense	c.3574 C>T	p.(Gln1192*)	LOVD	
Exon 31	Duplication	c.3682dup	p.(Leu1228Profs*6)	**This study**	

Exon 31	Deletion	c.3712_3715del	p.(Ala1238Serfs*86)	**This study**	
Exon 34	Nonsense	c.4037C>A	p.(Ser1346*)	[1]	
Exon 34	Insertion	c.4145_4146insC	p.(Ser1383Glufs*31)	**This study**	
Exon 34	Deletion	c.4258_4261del	p.(Ser1420Glyfs*55)	[1]	
Exon 34	Deletion	c.4279del	p.(Ser1427Valfs*49)	[1]	
Exon 34	Delins	c.4315_4326delinsCT	p.(Gly1439Leufs*67)	**This study**	
Exon 34	Nonsense	c.4406C>G	p.(Ser1469*)	LOVD	
Exon 35	Deletion	c.4535_4539del	p.(Asp1512Valfs*10)	**This study**	
Exon 35	Nonsense	c.4537G>T	p.(Glu1513*)	LOVD	
Exon 36	Nonsense	c.4606C>T	p.(Gln1536*)	[1]	
Exon 36	Missense	c.4662G>T	p.(Gln1554His)	[1]	Not present in gnomAD. Reported as de novo (LOVD). FA: Disrupts function [1]
Exon 37	Missense	c.4672G>A	p.(Glu1558Lys)	[1]	Not present in gnomAD. Reported as de novo (LOVD). **FA: Disrupts function (this study)**
Exon 37	Missense	c.4708A>T	p.(Arg1570Trp)	[1]	Not present in gnomAD FA: Disrupts function (LOVD) [1]
Exon 37	Nonsense	✦c.4779C>G	p.(Tyr1593*)	[1]	
Exon 38	Missense	c.4858C>T	p.(His1620Tyr)	[1]	Not present in gnomAD. Reported as *de novo*[1]
Exon 38	Deletion	c.4912_4914del	p.(Lys1638del)	[8]	Not present in gnomAD. Reported as *de novo* multiple times (LOVD).
Exon 38	Deletion	c.4925del	p.(Gly1642Alafs*30)	[1]	
Exon 38	Missense	c.4925G>A	p.(Gly1642Asp)	[17]	Not present in gnomAD FA: Disrupts function (LOVD) [17]
Exon 38	Missense	c.4958C>T	p.(Ser1653Phe)	[19]	Not present in gnomAD FA: Disrupts function (LOVD) [19]
Exon 39	Nonsense	c.4993C>T	p.(Gln1665*)	LOVD	Reported as de novo (LOVD).
Exon 39	Missense	c.5043C>G	p.(Asn1681Lys)	[20]	Not present in gnomAD. Reported as de novo (LOVD).**FA: Disrupts function (this study)**

Exon 39	Missense	✦c.5024C>T	p.(Pro1675Leu)	[11]	Not present in gnomAD. Reported as de novo multiple times (LOVD). Disrupts function [19]
Exon 39	Deletion	c.5053del	p.(Leu1685Cysfs*141)	http://tsc-project.partners.org/	
Exon 39	Insertion	c.5059_5060insT	p.(Cys1687Leufs*19)	**This study**	
Exon 39	Deletion	c.5065_5068+1del	p.(Lys1689Thrfs*136)	**This study**	
Exon 40	Duplication	c. 5116_5119dup	p.(Asn1707Thrfs*23)	**This study**	
Exon 40	Missense	✦c.5126C>T	p.(Pro1709Leu)	[10]	Not present in gnomAD. Disrupts function (LOVD)[19]
Exon 40	Deletion	c.5136_5137del	p.(Arg1713Profs*15)	[1]	
Exon 41	Nonsense	c.5208C>A	p.(Tyr1736*)	[1]	
Exon 41	Deletion	c.5212del	p.(Ser1738Profs*88)	**This study**	
Exon 41	Nonsense	c.5220G>A	p.(Trp1740*)	[21]	
Exon 41	Missense	c.5227C>T	p.(Arg1743Trp)	[1,22]	Not present in gnomAD. Disrupts function (LOVD) [17]
Exon 41	Missense	✦✦✦c.5228G>A	p.(Arg1743Gln)	[1]	Not present in gnomAD. Disrupts function LOVD [17]
Exon 41	Deletion	✦✦✦✦c.5238_5255del	p.(His1746_Arg1751del)	[1,10]	Not present in gnomAD. Disrupts function LOVD.[17]
Intron 6	Splicing	c.600-2A>G	p?	[1,10]	Not present in gnomAD. Predicted change at acceptor site 2 bps downstream:-100.0%
Intron 6	Splicing	✦c.599+5G>A	p?	LOVD	Not present in gnomAD. Predicted change at donor site 5 bps upstream: -99.6%
Intron 9	Splicing	c.849-3T>G	p?	[1]	Not present in gnomAD. Predicted change at acceptor site 3 bps downstream:+100.0%
Intron 11	Splicing	c.1120-2A>G	p?	**This study**	Not present in gnomAD. Predicted change at acceptor site 2 bps downstream: 100.0%
Intron 12	Splicing	c.1258-2delA	p?	**This study**	Not present in gnomAD. Predicted change at acceptor site 2 bps downstream: 100.0%

Intron 13	Splicing	c.1362-2A>G	p?	[1]	Not present in gnomAD. Predicted change at acceptor site 2 bps downstream: 100.0%
Intron 14	Splicing	c.1444-1G>T	p?	LOVD	Not present in gnomAD. Predicted change at acceptor site 1 bps downstream:-100.0%
Intron 13/ Exon 14	Delins	c.1362-63_1382delinsCAG	p?	**This study**	Not present in gnomAD. Predicted change at acceptor site 0 bps upstream: -100.0%
Intron 15	Splicing	c.1600-1G>T	p?	**This study**	Not present in gnomAD. Predicted change at acceptor site 1 bps downstream: -100%
Intron 27	Splicing	c.3132-2A>C	p?	[1]	Not present in gnomAD. Predicted change at acceptor site 2 bps downstream: -100.0%
Intron 28	Splicing	c.3284+1G>C	p?	[23]	Not present in gnomAD. Predicted change at donor site 1 bps upstream:- 100.0%
Intron 30	Splicing	3611-2A>G	P?	LOVD	Not present in gnomAD. Predicted change at acceptor site 2 bps downstream:- 100.0%
Intron 33	Splicing	4005+1G>C	P?	LOVD	Not present in gnomAD. Predicted change at donor site 1 bps upstream: -100%
Intron 36	Splicing/deletion	c.4663-27_4668del	p?	**This study**	Not present in gnomAD Predicted change at acceptor site 0 bps upstream: -100.0%
Intron 39	Splicing	c.5069-1G>T	p?	[1]	Not present in gnomAD. Predicted change at acceptor site 1 bps downstream: -100%
Intron 41	Splicing	c.5160+5G>A	p?	[1]	
Exon 4	Deletion	Ex4del	c.226-?_336+?	[1]	
Exon 2-10	Deletion	Ex2_10del	c.1-?_975+?	**This study**	
Exon 14	Deletion	Ex14del	c.(1361+1_1362-1)_(1443+1_1444-1)del	**This study**	
Exon 15	Deletion	Ex15del	c.(1443+1_1444-1)_(1599+1_1600-1)del	LOVD	

Exon 16	Deletion	Ex16del	c.(1599+1_1600-1)_ (1716+1_1717-1)del	LOVD	
Exon 17-29	Deletion	Ex17_29del	c.(1716+1_1717-1)_ (3397+1_3398-1)del	**This study**	
Exon 17-30	Deletion	Ex17_30del	c.(1716+1_1717-1)_ (3610+1_3611-1)del	[24]	
Exon 31-42 (+PKD1)	Deletion	c.3611-?_5424+?	c.(3610+1_3611-1)_?	[25]	
Entire gene	Deletion	✦ Gene deletion	c.(-107+1_3611-1)_?	[24]	
**VARIANTS IN TSC2 PREDICTED BENIGN, LIKELY BENIGN OR OF UNCERTAIN PATHOGENICICY**					
**Position**	**Coding effect**	**Mutation**	**Annotation**	**Reference**	**Notes**
Exon 10	Missense Benign	#c.856A>G	p.(Met286Val)	[9]	gnomAD, All: 0.18% East Asian: 1.9% **FA: No effect (this study)**
Exon 18	Missense Both Likely benign	c.1915C>T/c.1292C>T	p.(Arg639Trp/p.Ala431Val)	LOVD	gnomAD. All: 0.0041% FA: **No effect (this study)**
Intron 5	Splicing Likely benign	c.336+14C>T	p?	**This study**	gnomAD, All:0.0040% Predicted effect on splicing: 0%
Intron 11	Splicing Uncertain pathogenicity	c.976-16C>A	p?	**This study**	Not present in glomAD. Predicted change at acceptor site 16 bps downstream: -38.5%
Intron 28	Splicing Uncertain pathogenicity	c.3284+3G>A	p?	**This study**	Not present in gnomAD. Predicted change at donor site 3 bps upstream: +68.4%
Intron 32	Splicing Uncertain pathogenicity	c.3883+5C>T	p?	[1]	Frequency in gnomAD: All:0.00080%. Predicted change at donor site 5 bps upstream: -11%
Intron 42	Splicing/deletion. Uncertain pathogenicity	c.5260-34_5260-10del	p?	**This study**	Not present in gnomAD Predicted change at acceptor site 10 bps downstream: - 8.9%

Supplementary Table 4. *TSC2* variants identified in Danish TSC patients during the period 2003-2018. Overview of predicted pathogenic or likely pathogenic *TSC2* variants. The lower part shows selected *TSC2* variants classified as benign, likely benign or of uncertain pathogenicity (VUS). *In silico* prediction of novel *TSC2* variants using Alamut including access to MaxEntScan[4], NNSPLICE[5], and Human Splice Finder[6] for predicting of the effect on splicing. Observed in two unrelated patients; ✦✦ Observed in three unrelated patients; ✦✦✦ Observed in four unrelated patients; ✦✦✦✦ Observed in five unrelated patients

References to supplementary Tables 3 and 4.

1. Rendtorff, N. D. *et al.* Analysis of 65 tuberous sclerosis complex (TSC) patients by *TSC2* DGGE, *TSC1* / *TSC2* MLPA, and *TSC1* long-range PCR sequencing, and report of 28 novel mutations. *Hum. Mutat.* **26**, 374–383 (2005).
2. Hoogeveen-Westerveld, M. *et al.* Functional assessment of variants in the TSC1 and TSC2 genes identified in individuals with Tuberous Sclerosis Complex. *Hum. Mutat.* **32**, 424–435 (2011).
3. van Slegtenhorst, M. *et al.* Identification of the tuberous sclerosis gene TSC1 on chromosome 9q34. *Science* **277**, 805–8 (1997).
4. Yeo, G. & Burge, C. B. Maximum Entropy Modeling of Short Sequence Motifs with Applications to RNA Splicing Signals. *J. Comput. Biol.* **11**, 377–394 (2004).
5. REESE, M. G., EECKMAN, F. H., KULP, D. & HAUSSLER, D. Improved Splice Site Detection in Genie. *J. Comput. Biol.* **4**, 311–323 (1997).
6. Desmet, F.-O. *et al.* Human Splicing Finder: an online bioinformatics tool to predict splicing signals. *Nucleic Acids Res.* **37**, e67–e67 (2009).
7. CHOY, Y. S. *et al.* Superiority of Denaturing High Performance Liquid Chromatography over single-stranded conformation and conformation-sensitive gel electrophoresis for mutation detection in TSC2. *Ann. Hum. Genet.* **63**, 383–391 (1999).
8. Au, K. S. *et al.* Genotype/phenotype correlation in 325 individuals referred for a diagnosis of tuberous sclerosis complex in the United States. *Genet. Med.* **9**, 88–100 (2007).
9. Jones, A. C. *et al.* Comprehensive Mutation Analysis of TSC1 and TSC2—and Phenotypic Correlations in 150 Families with Tuberous Sclerosis. *Am. J. Hum. Genet.* **64**, 1305–1315 (2002).
10. Dabora, S. L. *et al.* Mutational analysis in a cohort of 224 tuberous sclerosis patients indicates increased severity of TSC2, compared with TSC1, disease in multiple organs. *Am. J. Hum. Genet.* **68**, 64–80 (2001).
11. Niida, Y. *et al.* Analysis of bothTSC1 andTSC2 for germline mutations in 126 unrelated patients with tuberous sclerosis. *Hum. Mutat.* **14**, 412–422 (1999).
12. Nellist, M. *et al.* TSC2 missense mutations inhibit tuberin phosphorylation and prevent formation of the tuberin-hamartin complex. *Hum. Mol. Genet.* **10**, 2889–2898 (2001).
13. Choi, J. E., Chae, J. H., Hwang, Y. S. & Kim, K. J. Mutational analysis of TSC1 and TSC2 in Korean patients with tuberous sclerosis complex. *Brain Dev.* **28**, 440–446 (2006).
14. Jansen, A. C. *et al.* Unusually mild tuberous sclerosis phenotype is associated with *TSC2* R905Q mutation. *Ann. Neurol.* **60**, 528–539 (2006).
15. Yamashita, Y. *et al.* Analysis of all exons oftsc1 andtsc2 genes for germline mutations in Japanese patients with tuberous sclerosis: Report of 10 mutations. *Am. J. Med. Genet.* **90**, 123–126 (2000).
16. Sancak, O. *et al.* Mutational analysis of the TSC1 and TSC2 genes in a diagnostic setting: genotype – phenotype correlations and comparison of diagnostic DNA techniques in Tuberous Sclerosis Complex. *Eur. J. Hum. Genet.* **13**, 731–741 (2005).
17. Langkau, N. *et al.* TSC1 and TSC2 mutations in tuberous sclerosis, the associated phenotypes and a model to explain observed TSC1/TSC2 frequency ratios. *Eur. J. Pediatr.* **161**, 393–402 (2002).
18. Hoogeveen-Westerveld, M. *et al.* Functional Assessment of *TSC* 2 Variants Identified in Individuals with Tuberous Sclerosis Complex. *Hum. Mutat.* **34**, 167–175 (2013).
19. Soucek, T. *et al.* Tuberous sclerosis causing mutants of the TSC2 gene product affect proliferation and p27 expression. *Oncogene* **20**, 4904–4909 (2001).

20. Roberts, P. S. *et al.* SNP identification, haplotype analysis, and parental origin of mutations in TSC2. *Hum. Genet.* **111**, 96–101 (2002).
21. Hung, C.-C. *et al.* Molecular and clinical analyses of 84 patients with tuberous sclerosis complex. *BMC Med. Genet.* **7**, 72 (2006).
22. Jang, M. A. *et al.* Identification of TSC1 and TSC2 mutations in Korean patients with tuberous sclerosis complex. *Pediatr. Neurol.* **46**, 222–224 (2012).
23. Longa, L. *et al.* TSC1 and TSC2 deletions differ in size, preference for recombinatorial sequences, and location within the gene. *Hum. Genet.* **108**, 156–166 (2001).
24. Oyazato, Y. *et al.* Molecular analysis of TSC2/PKD1 contiguous gene deletion syndrome. *lib.kobe-u.ac.jp*
